# Exploring the pretraining effect in learning classical Chinese reading skills

**DOI:** 10.3389/fpsyg.2026.1692135

**Published:** 2026-02-10

**Authors:** Dayu Jiang, Jing Zhou, Mengyao Ma, Slava Kalyuga

**Affiliations:** 1 School of Foreign Languages and Literature, Wuhan University, Wuhan, China; 2 School of Foreign Studies, Zhongnan University of Economics and Law, Wuhan, China; 3 School of Education, University of New South Wales, Sydney, NSW, Australia

**Keywords:** classical Chinese reading skills, cognitive load theory, expertise reversal effect, instructional design, pretraining effect

## Abstract

Reading Chinese classical texts is very challenging, even for native speakers of Chinese. This study investigated the pretraining effect (established in cognitive theory of multimedia learning and cognitive load theory) in reading classical Chinese texts by young learners of Chinese as the first language. A 3 × 2 factorial design was adopted with pretraining activities modes (thematic pretraining, linguistic pretraining, and no pretraining) and levels of expertise (two levels of relative proficiency in classical Chinese reading comprehension) as independent variables and with reading comprehension performance and cognitive load ratings as dependent variables. 129 Year-6 (less proficient) students and 151 Year-8 (more proficient) students were randomly allocated to three instructional conditions. It was found that the more proficient Chinese learners in the two pretraining groups outperformed the more proficient learners in the no pretraining group, and the thematic pretraining condition led to better learning outcomes and lower cognitive load ratings than the linguistic pretraining condition. For the less proficient participants, the thematic pretraining condition had lower cognitive load ratings and better learning outcomes than no pretraining conditions. The results were discussed from the perspective of cognitive load theory.

## Introduction

Classical Chinese learning is “a core component of the Chinese language curriculum in all Chinese society” (Lau, 2018, p. 140). Reading classical Chinese is also essential to learning Chinese culture and history, as a large number of Chinese documents were written in the genre of classical Chinese. However, learning to read classical Chinese can be very challenging (Lau, 2004; Lau and Qian, 2025; Lin and Li, 2023), especially for young native speakers of Chinese, as “students often find classical Chinese texts obscure and difficult to understand” (Wu and Chen, 2018, p. 65). On top of this, reading itself is particularly complicated as it “involves language knowledge and cognitive processes at the word, sentence, and discourse levels,” which associates with “the process of decoding and comprehension” (Lau, 2018, p. 139). From a linguistic perspective, classical Chinese reading essentially involves the acquisition of three types of knowledge: linguistic knowledge, literary knowledge and cultural knowledge (Lau, 2018). The intertwined nature of these elements in classical Chinese learning may impose heavy working memory load on learners.

The pretraining effect or principle is a strategy for learners to manage working memory load when learning complex instructional contents (i.e., materials with high element interactivity which contain many interrelated elements of information that need to be processed at the same time) (Mayer et al., 2002; Sweller et al., 2011). Mayer et al. (2002) proposed a two-stage approach to reduce cognitive load and facilitate learning outcomes. The first stage (pretraining) involves instruction in isolated individual components and their respective characteristics, while the second stage consolidates the key concepts and terms introduced in the first stage and considers all essential interactions between them. A similar technique, called isolated-interacting elements effect, was also suggested in cognitive load theory (see Sweller et al., 2011, for an overview of the technique).

Despite the growing interests in the field of classical Chinese learning, the number of empirical research studies investigating classical Chinese reading comprehension and learning is still limited (Lau, 2018). To our best knowledge, no empirical research has been conducted to investigate the pretraining effect in classical Chinese learning from the perspective of cognitive load theory. A thorough understanding of a classical Chinese work requires readers to understand its thematic features, linguistic components, and cultural elements. This research aims to establish whether providing thematic and linguistic information as ways of pretraining could facilitate the acquisition of classical Chinese language skills.

### Classical Chinese reading as a complex cognitive task

It is generally acknowledged that classical Chinese texts are high element interactivity materials (i.e., they involve many interconnected elements of information that need to be process concurrently in reader working memory to make sense of a text), thus creating great challenges to many students (Lau, 2018; Lau and Qian, 2025). Although students can translate the majority of classical Chinese terms into modern Chinese or Mandarin, they may still find it difficult to fully comprehend the texts not only because the classical Chinese texts were written hundreds or thousands of years ago, but also because classical Chinese is different from modern Chinese at lexical, grammatical, and discursive levels.

For example, most Chinese characters are compound characters composed of both semantic and phonetic components. Lexical compounding is particularly common in Chinese, and grammatical information is relatively less marked and explicit in Chinese sentences (Chang et al., 2022; Ku and Anderson, 2003; McBride-Chang et al., 2010; Shu and Anderson, 1997; Zhang et al., 2014). Vocabularies in classical Chinese texts differ from modern Chinese: classical Chinese words are generally monosyllabic rather than multisyllabic and always have multiple meanings (Wang, 1979; Wang et al., 2007; Wei, 2009; Zhang, 2005). These differences make word recognition a major problem in students’ classical Chinese reading comprehension.

Therefore, classical Chinese reading relies substantially on lower-level skills. At the sentence level, classical Chinese’s basic structure is very similar to modern Chinese. Two types of classical Chinese sentences are frequently considered difficult to understand: elliptical sentences with omitted components, and inverted sentences wherein word orders differ from normal sentences (Pulleyblank, 1995). At the discourse level, previous research studies (e.g., Ozuru et al., 2008; Pressley and Afflerbach, 1995) support the claim that effective and in-depth comprehension depends on sufficient prior knowledge of a given reading topic and the ability to activate appropriate schemas.

### Cognitive load theory and its implication for classical Chinese Reading

Cognitive load theory is an instructional theory based on our knowledge of the features of human cognitive architecture and its evolutionary foundation (Jiang, 2024; Jiang et al., 2018; Sweller et al., 2011; Sweller et al., 1998; van Merriënboer and Sweller, 2005). This theory throws light on whether pretraining activities could facilitate classical Chinese reading comprehension, a cognitive task with high levels of element interactivity. Cognitive load theory suggests two kinds of cognitive load: intrinsic and extraneous (Jiang, 2024; Jiang and Kalyuga, 2020; Sweller et al., 2011). Intrinsic cognitive load is generated by the complexity of instructional materials, which is determined by their associated levels of element interactivity (Jiang et al., 2023). Highly interactive and interdependent elements of information need to be processed simultaneously in working memory in order to construct a corresponding schema and achieve understanding, thus imposing a heavy intrinsic cognitive load.

For example, while reading a piece of classical Chinese work, learners need to process and integrate literal meaning, thematic connotation and cultural background simultaneously. Novice learners without or with little prior knowledge may need to devote a substantial part of their cognitive resources to understanding literal meaning in the text. As a result, their limited working memory may not process all the information needed for successful comprehension (Lee and Kalyuga, 2011; Kalyuga et al., 2013; McLaughin, 1990). However, the complexity of learning materials always depends on learner levels of expertise (Jiang et al., 2018; Kalyuga, 2006, 2007; Kalyuga et al., 2000). Classical Chinese texts which are regarded as high element interactivity materials for novice Chinese learners may not impose heavy cognitive load on higher proficiency learners.

Extraneous cognitive load is referred to the type of working memory load irrelevant to essential learning. It is generated by learner activities that are not related to schema construction and automation (Jiang et al., 2017; Sweller et al., 1998). For example, some learning materials may be designed in a way that requires learners to mentally integrate different sources of information, for instance, a text with separate vocabulary definitions. Such integration may require learners to devote additional cognitive resources to retaining the relevant segments of text in working memory while searching and matching corresponding vocabulary definitions. This split attention situation could be eliminated by physically integrating the vocabulary definitions into the text (Yeung et al., 1998).

The main concern of cognitive load theory is to increase the efficiency of learning. The theory suggests that whereas intrinsic cognitive load should be properly managed (e.g., by selecting learning tasks that match levels of learner prior knowledge), extraneous cognitive load should always be minimized in order to guarantee the total load does not exceed the capacity of working memory (Paas et al., 2004). An excessive extraneous cognitive load due to sub-optimal instructional designs may leave insufficient cognitive resources required for learning the essential knowledge. A number of cognitive load effects have been generated to optimize the amount of cognitive load and to promote learning, among which are the pretraining effect and the expertise reversal effect. These two effects are relevant to the reported research study and will be reviewed next.

### Pretraining effect

The pretraining effect is a strategy for managing cognitive load, particularly intrinsic cognitive load, in instruction by providing fundamental knowledge points of key components (usually in the forms of terms and characteristics of the main concepts) prior to a lesson to ensure the mastery of core instructional information (Fiorella and Mayer, 2012; Mayer, 2021; Pilegard and Mayer, 2016, 2018; Pollack et al., 2002). Even though pretraining activities have been adopted in classrooms for a long time, pretraining effect was first discussed by Mayer et al. (2002). In their studies, the participants were required to learn the functions of a car’s braking system or a bicycle tire pump from a narrated animation and then take problem-solving transfer tests. Before watching the animation, the participants in the pretraining group learned the terms and possible states of each part in the car’s brake system through an interactive computer tutorial. Similarly, before the tire pump lesson, the students in the pretraining group were provided with a clear plastic model of the pump, from which they could see the states of individual components when they pulled up and pushed down the handle. On the subsequent delayed test of problem-solving transfer, the participants in the pretraining group outperformed the students in the control group in three experiments.

Evidence on the pretraining effect has been accumulating. Mayer et al. (2002) found out that the participants who learned the illustrations of the major geological terms prior to the lesson had better problem-solving performance in the post-test than the participants who did not learn the pretraining materials. In Gegner et al.’s (2009) study, the students in the pretraining group had been provided with the background information of the teaching contents before the instruction had better comprehension test scores than the students who had not received pretraining materials. These research findings were echoed by Sanchez and Reber (2013) who argued that “explicit pretraining instruction led to robust explicit knowledge” (p. 341).

### Linguistic pretraining and thematic pretraining in language teaching

In the field of language teaching, pretraining activities have been provided in the form of pre-teaching or pre-learning for a long time, though this instructional technique has not yet been investigated explicitly through the lens of cognitive load theory. Reading is a complex cognitive activity involving multiple types of knowledge and skills as well as sophisticated cognitive processing at different levels (Grabe, 2009). Presenting some essential elements in the pretraining phase could reduce the number of interactive elements in working memory during formal learning. For example, Rondon and Tomitch (2023) argued that providing pretraining supports in the pre-reading stage could ‘reduce processing demands in working memory” (p. 1). Berg and Wehby (2013) recommended two types of pretraining strategies: teaching new vocabulary prior to a lesson that students could revisit during the session and providing a thematic framework or schema for the upcoming teaching. Accordingly, preteaching vocabulary could facilitate the activation of lower-level language skills while preteaching thematic knowledge fosters the application of high-level language skills within the framework of multi-component view of reading comprehension (Calloway et al., 2023; Hogan et al., 2011; Lau, 2018; Perfetti et al., 2005; Silva and Cain, 2015; Watson et al., 2012).

Lower-level language skills include word identification, meaning retrieval, and grammatical computation, which is crucial for understanding the words and sentences in a text. Classical Chinese texts demand specific lower-level skills, such as radical awareness, syntactic knowledge, the use of semantic clues, and morphological skills, due to the distinctive nature of Chinese characters, compound characters, and the relatively less explicit grammatical markers in Classical Chinese (Lau, 2018). Higher-level language skills, such as inferencing, comprehension monitoring, and using prior knowledge, enable readers to construct an integrated representation of the text meaning, often referred as a mental model (Calloway et al., 2023). Reading Classical Chinese involves additional higher-level skills, as readers must integrate not only linguistic knowledge but also cultural and historical context to fully comprehend the text. This makes reading Classical Chinese even more complex, as it requires a broader range of cognitive and metacognitive skills compared to reading in modern Chinese or European languages.

Following this line, previous studies on pretraining in language teaching were reviewed from two perspectives: linguistic pretraining and thematic pretraining. Linguistics pretraining involves the provision of foundational linguistic knowledge, such as vocabulary and grammar, with a focus on developing lower-level language skills, prior to formal instructional sessions. This instructional technique aims to equip learners with the basic linguistic structures and lexical items necessary for engaging with more complex language tasks, thus laying the groundwork for subsequent, more advanced language learning activities. It has been reported that learners experienced great difficulties with vocabularies in classical Chinese reading (Lau, 2018; Lau and Qian, 2025). Huang et al. (2006) reported that providing vocabulary list as a pre-reading activity could directly facilitate learners’ academic English comprehension. Webb (2009, 2010) collectively examined the impact of pretraining vocabulary on language learning outcomes. In both studies, he found that different approaches to vocabulary pretraining, whether through receptive or productive tasks, or by prelearning low-frequency words, could enhance both reading comprehension and writing skills.

The benefits of linguistic pretraining were also evident in Love et al.’s (2017) study, which reported that using E-books as a tool to preteach key academic vocabulary could facilitate “students’ access to content standards and their overall success in general education content courses” (p. 92). Moreover, Tajeddin and Mansouri (2024) reported that language teachers often taught essential language knowledge including vocabulary and grammar in advance to help learners perform well in teaching tasks. Yekta et al. (2024) conducted an empirical study to compare how inductive and deductive aural vocabulary pretraining could affect English as a foreign language learners’ word recognition from speech. It was found the two pretraining instructional groups significantly outperformed the control group, clearly demonstrating the pretraining effect.

In this study, thematic pretraining refers to the process of introducing literary and/or cultural knowledge that emphasizes higher-order cognitive skills, prior to engaging in formal language instruction. This approach aims to provide learners with a contextual and conceptual foundation, thereby enhancing their understanding and application of language within specific thematic frameworks. By focusing on thematic content, such pretraining helps bridge cultural and intellectual knowledge with language acquisition, which fosters deeper comprehension and more meaningful language learning experiences.

For example, McVicker (2007) acknowledged the beneficial role of comics as a text structure to help children read as “the visual representation of the text provides the reader with a deeper comprehension of the author’s intended message” (p. 87). Dong et al.’s (2025) study highlighted the critical role of pre-reading training in enhancing children’s topic background knowledge, which significantly supports language development and reading outcomes. By providing relevant or irrelevant prelearning activities, the researchers showed that thematic pretraining effectively improved reading comprehension, vocabulary, and reduced reading anxiety during formal reading interventions. Tseng’s (2025) study found that teachers usually adopted visual scaffolding as a pretraining technique in that visual scaffolding “improves the comprehensibility of English scientific materials for EFL students” (p. 1).

### Expertise reversal effect and pretraining effect

Research has also found that learners’ expertise mediates the pretraining effect (Jiang et al., 2018). Pollack et al. (2002) reported that less-experienced participants benefited more from pretraining activities than more-experienced learners in an electrical engineering course. In another set of early studies, Clarke et al. (2005) provided students with limited spreadsheet skills with pretraining activities on key features of the spreadsheet. It was found that the low-expertise learners who received pretraining demonstrated relatively better mathematics learning by using spreadsheet software than the high-expertise students. These two studies manifested the expertise reversal effect: low-expertise learners gained significant benefits from the pretraining activities but high-expertise learners did not. The reason might be that high-expertise learners perceived the individual terms and characteristics redundant as they already had these knowledge structures, while low-expertise learners found these pretraining information essential as they did not have these knowledge structures ready in their long-term memories.

Liu (2004) investigated whether the absence or presence of comic strips with texts of high/low difficulty levels would affect high-proficiency and low-proficiency learners’ reading comprehension. It was found that providing comic strips with more complex texts facilitated low-proficiency learners’ performance compared to providing the texts only. A related recent study by Yao et al. (2025) found that providing specific illustrations corresponding to each line of a poem significantly benefited lower-expertise Chinese speakers’ reading comprehension of poetry texts than providing only one general illustration for the whole poem. Complementing these quantitative results, qualitative interviews-based data also showed that the multiple illustrations assisted the learners in constructing the thematic plots of the poem. However, Woolley (2014) argued that skilled readers did not simply retain verbal information but develop more dynamic knowledge structures “by constructing a mental model incorporating both visual and verbal information in the form of a cohesive representation of the meaning” (p. 215). This indicates that providing visual scaffolding in the form of thematic pretraining might benefit higher-expertise learners.

Even though earlier studies show that learners’ expertise may be a contributing factor to the pretraining effect, to our best knowledge, no research has been conducted to investigate the influence of learner expertise on the pretraining effect in classical Chinese reading comprehension. This study aimed to investigate how pretraining activities (linguistic and thematic) could affect relatively high- and low-expertise young Chinese learners’ classical Chinese reading comprehension. According to the pretraining effect, the meaning of individual characters in classical Chinese texts could be used as linguistic pretraining materials, which could promote learners’ learning of the literal meaning (classical Chinese), thematic meaning (literature) and cultural knowledge (culture) in formal instruction. However, a blank prescription with a list of key terms and definitions for the learner may not be an optimal and effective form of pretraining. Another form of pretraining was implemented in this study – thematic pretraining. Thematic pretraining can be a set of comic strips or cartoons depicting the general plot of the classical Chinese text. It is assumed that visual information could help learners establish connections between linguistic information and thematic meaning, thus facilitating their comprehension of the classical Chinese text. In previous studies (e.g., Clarke et al., 2005; Pollack et al., 2002), the pretraining effect was obtained for less-experienced learners but not for more-experienced learners. Therefore, this study attempted to explore the following research questions.

*Research Question 1*. How do different kinds of pretraining (thematic pretraining, linguistic pretraining, and no pretraining) affect the reading comprehension of classical Chinese texts by learners with higher prior knowledge and lower prior knowledge?

*Research Question 2*. How do different kinds of pretraining (thematic pretraining, linguistic pretraining, and no pretraining) affect the cognitive load experienced by learners with higher prior knowledge and lower prior knowledge?

## Method

The Humanities and Social Sciences Ethics Advisory Panel at Wuhan University, China, granted approval to carry out the current research.

### Research design

This study adopted a quasi-experimental quantitative research design, with instructional approach as an independent variable and learning performance and cognitive load ratings as dependent variables. Such designs have been widely employed in language teaching and applied linguistics research, particularly in intervention research studies conducted in authentic instructional settings (Dörnyei, 2007). For example, Huang and Zhang (2020) as well as Li and Zhang (2022) adopted quasi-experimental designs to investigate instructional effects in language learning contexts.

**Figure 1:**
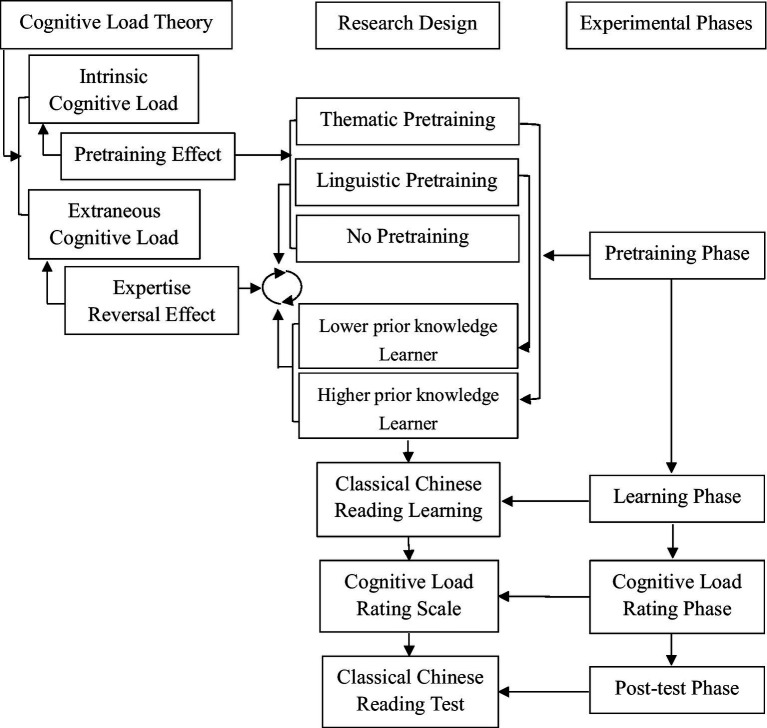
An overview of conceptual framework, research design, and experimental phases.

Figure 1 provides an overview of the conceptual framework, research design, and experimental phases. The theoretical framework of the present study was grounded in Cognitive Load Theory. Based on the pretraining principle, pretraining was expected to alleviate learners’ intrinsic cognitive load. Three instructional approaches were examined: thematic pretraining, linguistic pretraining, and no pretraining. In addition, the expertise reversal effect suggests that the effectiveness of instructional support varies according to learners’ expertise level, often due to changes in extraneous cognitive load. Accordingly, an interaction between pretraining approaches and learner expertise was hypothesized, such that thematic pretraining would be more beneficial for higher prior knowledge learners, whereas linguistic pretraining would be more beneficial for lower prior knowledge learners in terms of learning outcomes and cognitive load. The experiment consisted of four phases: pretraining, instruction, cognitive load rating, and post-test.

### Participants

A convenience sampling method was adopted to recruit participants. Year 6 students from three intact classes from a public primary school in central China and Year 8 students from three intact Year 8 classes from a public secondary school in central China participated in the study. As Year 8 students had received approximately two additional years of formal instruction in classical Chinese compared with the Year 6 students, they were considered to have relatively higher proficiency and broader prior knowledge in Classical Chinese reading comprehension. In this study, proficiency was operationalized as relative differences in prior knowledge resulting from differential instructional experience.

Invitation letters and informed consent forms were provided to all prospective participants and their parents or legal guardians. The materials explained the research aims, research procedures, potential risks, participants’ rights to withdraw from the research at any time without penalty, measures for ensuring confidentiality, and the contact information for the researchers. Written informed consent was obtained from parents or guardians, and assent was obtained from all participating students prior to data collection. In total, one hundred and twenty-nine Year 6 students and one hundred and fifty-one Year 8 students returned the signed consent forms and voluntarily participated in the research. Among the participants, 139 were female. The mean age of the less proficient group was 12 years, while the mean age of the more proficient group was 14 years.

Three intact classes at each year level were randomly assigned to three instructional conditions: no pretraining (45), linguistic pretraining (39), and thematic pretraining (45) for Year 6; no pretraining (48), linguistic pretraining group (53), and thematic pretraining (50) for Year 8. All three classes at each level had the same teacher of Classical Chinese, thus eliminating a possible teacher effect. The scores of the participants’ latest semester-end tests were used to compare the levels of prior knowledge in different experimental conditions at each educational level, respectively. A one-way ANOVA showed that there were no significant differences in pre-test scores among the Year 6 participants in the three instructional conditions, *F*(2, 126) = 0.33, *p* = 0.72. Similarly, a one-way ANOVA found no significant differences in the pre-test scores among Year 8 participants in the three experimental conditions, *F*(2, 148) = 0.31, *p* = 0.73.

## Materials and procedures

A piece of classical Chinese text titled *Duke Wang audited literature and history* (王荆剬旁听文史; *Wang Jinggong Pang Ting Wen Shi*) was chosen for the instructional materials. This text was not included in the Chinese syllabus of these year levels, so it could be regarded as novel information for the participants. The instructional materials were reviewed by two experienced Chinese teachers and one expert in the field of educational testing and instructional design to ensure that the experimental materials were appropriate to the participants at the selected year levels. The experiment was conducted in normal classroom settings in a session of 45 min, including pretraining phase (5 min), learning phase (18 min), cognitive load rating phase (4 min), and post-test phase (18 min).

### Pretraining phase

The students in linguistic pretraining (LP) groups were asked to study the LP material, which contained 13 key terms, their corresponding pinyin, and their modern Chinese translations, with the purpose of helping the students complete the sound-form-meaning mapping of the essential linguistic points and facilitating the bottom-up processing. The students in the thematic pretraining (TP) conditions were required to study a comic strip of six images depicting the general plot of the text. The thematic pretraining materials were intended to assist learners’ top-down processing. The students in no pretraining groups (NP) were presented with the learning material instead of the pretraining material. However, they did not receive any explicit instructions on how to proceed with the learning material in the pretraining phase. The pretraining phase, which lasted five minutes, was intentionally brief to align with classroom constraints and to minimize disruptions to regular instruction.

### Learning phase

The learning phase was conducted immediately after the pretraining phase. There were three learning tasks. First, the participants learned about the contextual knowledge in two minutes. The second task required the participants to learn the key Chinese characters and their corresponding pinyin in four minutes. The third task was text learning, in which the participants were asked to read and comprehend the text, and to translate the text into Mandarin. The text was put in the middle of the screen; the essential linguistic elements were underlined and lined to their corresponding explanatory notes in text boxes on two sides. The embedded format of information presentation aimed to alleviate any potential split-attention issues (Plass et al., 2003; Yeung et al., 1998).

### Cognitive load rating phase

After the learning phase, the students were asked to rate the levels of their cognitive load during learning. The cognitive load rating questionnaire (see Appendix 1), which was based on Leppink et al. (2013), included five items on intrinsic cognitive load and five items on extraneous cognitive load. This instrument has been widely used in research studies in the field of cognitive load theory (Jiang et al., 2018, 2020; Jiang and Kalyuga, 2020). The reliability of the subjective cognitive load rating scale as measured by Cronbach’s alpha was 0.91, indicating a high degree of reliability. Considering that the research participants were young learners, a five-point scale was used in order to help the participants make prompt and proper ratings.

### Post-test phase

The experiment ended with a post-test, which had five parts. In the first part, the students were asked to fill a Chinese character in the blank. The second question required the students to write pinyin for four Chinese characters. For the third question, the students needed to provide the meanings of four Chinese characters and phrases. The first three questions assessed learners’ lower-order reading knowledge. As for the fourth question, the learners were asked to summarize the main plot of the Classical Chinese text using no less than eighty words. The fifth question asked the students to make inferences about the theme of the classical Chinese text (i.e., what the author tries to teach in this literary text) and provide examples and evidence to support their ideas (i.e., how they come up with their conclusion). The last two tasks aimed to evaluate the learners’ higher-order reading skills. The maximum score of the post-test was 100 of which the vocabulary battery accounted for 28, the summative question for 42, and the inferential part for 30. Two independent raters assessed the participants’ post-test answers according to a marking scheme. The mean value of the two raters’ markings was used as the final score of the post-test. The Pearson intra-class correlation coefficient was 0.94, indicating a high degree of inter-rater reliability.

## Results

A 3 × 2 independent group factorial experimental design was used to compare the effectiveness of three pretraining methods (linguistic pretraining, thematic pretraining, no pretraining) for students with two levels of prior knowledge (higher and lower prior knowledge participants) using the classical Chinese comprehension test score and subjective rating of cognitive load as two dependent variables. Analysis of variances (ANOVA) was used to analyze the data. Means and standard deviations for the classical Chinese comprehension scores and the subjective ratings of cognitive load for the six groups (three instructional conditions at two levels of expertise) are provided in Table 1.

**Table 1:** Means and standard deviations for the total post-test, vocabulary part, summary part and inferential part scores, ratings of cognitive load, and instructional efficiency for different experimental conditions and learners’ levels of prior knowledge.

Expertise level	Lower prior knowledge			Higher prior knowledge		
Experimental condition	NP *n* = 45	LP *n* = 39	TP *n* = 45	NP *n* = 48	LP *n* = 53	TP *n* = 50
Post-test score						
*M*	63.20	70.18	74.24	75.38	83.89	87.18
SD	17.46	9.47	9.24	14.11	8.70	10.37
Vocabulary sub-test score						
*M*	20.40	24.23	24.98	22.42	25.70	25.00
SD	6.11	3.04	2.84	5.81	2.44	3.41
Summary sub-test score						
*M*	31.42	34.44	35.80	34.90	36.51	37.96
SD	6.97	3.64	3.99	5.55	4.25	3.34
Inferential sub-test score						
*M*	11.38	11.51	13.47	18.06	21.68	24.22
SD	7.36	5.26	4.87	5.53	6.81	5.28
Intrinsic cognitive load						
*M*	1.89	1.92	1.85	2.41	1.68	1.34
SD	0.76	0.64	0.81	1.03	0.70	0.47
Extraneous cognitive load						
*M*	1.72	1.95	1.78	2.23	1.54	1.37
SD	0.87	0.80	0.78	1.02	0.62	0.51
Total cognitive load						
*M*	1.80	1.94	1.82	2.32	1.61	1.36
SD	0.73	0.65	0.72	0.99	0.58	0.45
Instructional efficiency						
*M*	−0.65	−0.43	−0.11	−0.52	0.55	0.95
SD	1.28	0.92	1.08	1.38	0.74	0.71

The maximum post-test score was 100. The maximum scores for the three components were: vocabulary 28, summary, 42, and inferential 30. The maximum subjective rating scores for the cognitive load rating was 5.

NP, no pretraining; LP, linguistic pretraining; TP, thematic pretraining.

### Total post-test scores

A two-way ANOVA showed a significant main effect of expertise for post-test scores, *F*(1, 274) = 81.32, *p* < 0.01, *^η^р*^2^ = 0.23. Higher prior knowledge learners (*M* = 82.27, SD = 12.16) performed significantly better on post-test than lower prior knowledge learners (*M* = 69.16, SD = 13.51). There was also a significant main effect for types of pretraining on post-test scores, *F*(2, 274) = 22.27, *p* < 0.01, *^η^р*^2^ = 0.14. Pairwise comparisons indicated that the learners in the TP conditions (*M* = 81.05, SD = 11.75) significantly outperformed learners in both the LP conditions (*M* = 78.08, SD = 11.27) and NP conditions (*M* = 69.48, SD = 16.88). The learners who were provided with the linguistic pretraining activities significantly outperformed learners who did not receive any pretraining activities. There was no significant interaction between the three experimental conditions and learners’ levels of prior knowledge, *F*(2, 274) = 0.09, *p* = 0.91.

#### Summary part scores

A two-way ANOVA showed a significant main effect for prior knowledge level on summary part scores, *F*(1, 274) = 20.00, *p* < 0.01, *^η^р*^2^ = 0.07. Higher prior knowledge learners (*M* = 36.48, SD = 4.59) showed a better performance on summary part than lower prior knowledge learners (*M* = 33.86, SD = 5.44). There was also a significant main effect for types of pretraining on summary part scores, *F*(2, 274) = 14.44, *p* < 0.01, *^η^р*^2^ = 0.10. The learners in the TP condition (*M* = 36.94, SD = 3.80) and the learners in LP condition (*M* = 35.63, SD = 4.11) significantly outperform the learners in the NP condition (*M* = 33.22, SD = 6.48), with the TP groups outperforming the LP groups. There was no significant interaction between the three instructional conditions and learners’ levels of prior knowledge, *F*(2, 274) = 0.62, *p* = 0.54.

#### Inferential test scores

A two-way ANOVA showed a significant main effect of learners’ prior knowledge on inferential test scores, *F*(1, 274) = 165.98, *p* < 0.01, *^η^р*^2^ = 0.38. Learners with higher prior knowledge (*M* = 21.37, SD = 6.40) performed significantly better than learners with lower prior knowledge (*M* = 12.15, SD = 5.99). There was also a main effect of pretraining approach for inferential test scores, *F*(2, 274) = 11.31, *p* < 0.01, *^η^р*^2^ = 0.08. The learners in the TP condition (*M* = 19.13, SD = 7.40) and the learners in the LP condition (*M* = 17.37, SD = 7.97) significantly outperformed the learners in the NP groups (*M* = 14.83, SD = 7.27). There were no significant differences between the TP and LP groups. There was a significant interaction between the three experimental conditions and learners’ levels of prior knowledge, *F*(2, 274) = 3.18, *p* < 0.05, *^η^р*^2^ = 0.02. A significant simple effect of pretraining approaches was found for higher prior knowledge learners, *F*(2, 274) = 13.23, *p* < 0.01, *^η^р*^2^ = 0.09. Following this significant effect, a Tukey HSD *post hoc* test showed that the higher prior knowledge learners in the NP group had significantly lower test scores than the higher prior knowledge learners in both the LP group (*p* < 0.01) and in the TP group (*p* < 0.01). There was no significant difference between the scores for the higher prior knowledge learners in the LP condition and the higher prior knowledge learners in the TP condition. No significant simple effect was found for lower prior knowledge learners in terms of inferential test scores, *F*(2, 274) = 1.71, *p* = 0.18.

#### Rating of cognitive load

A two-way ANOVA showed no significant main effect of prior knowledge on the ratings of total cognitive load, *F*(1, 274) = 1.18, *p* = 0.28, *^η^р*^2^ = 0.004. There was a main effect of pretraining approaches on ratings of cognitive load, *F*(2, 274) = 10.86, *p* < 0.01, *^η^р*^2^ = 0.07. The TP groups (*M* = 1.57, SD = 0.64) and the LP groups (*M* = 1.75, SD = 0.63) had lower ratings of overall cognitive load than the NP groups (*M* = 2.07, SD = 0.91). In addition, the participants in the TP conditions reported lower levels of cognitive load than the participants in the LP instructional conditions. There was a significant interaction between the pretraining conditions and learners’ prior knowledge level, *F*(2, 274) = 13.12, *p* < 0.01, *^η^р*^2^ = 0.09. A significant simple effect was found for the learners with higher prior knowledge, *F*(2, 274) = 24.56, *p* < 0.01, *^η^р*^2^ = 0.15. Following this effect, a Tukey HSD *post hoc* test showed the higher prior knowledge learners in the no pretraining group had significantly higher ratings of total cognitive load than the higher prior knowledge learners in the linguistic pretraining group (*p* < 0.01) and higher prior knowledge learners in the thematic pretraining group (*p* < 0.01). However, the learners with higher prior knowledge in the linguistic pretraining group and the thematic pretraining group did not have significantly different ratings of total cognitive load. No significant simple effect was found for the learners with lower prior knowledge, *F*(2, 274) = 0.46, *p* = 0.63.

A two-way ANOVA found a significant main effect of pretraining approaches on the ratings of intrinsic cognitive load, *F*(2, 274) = 12.63, *p* < 0.01, *^η^р*^2^ = 0.08, indicating a small effect size. Both the learners in the TP conditions (*M* = 1.59, SD = 0.70) and the learners in the LP conditions (*M* = 1.78, SD = 0.68) had significantly lower ratings of intrinsic cognitive load than the learners in the NP conditions (*M* = 2.16, SD = 0.94). No significant difference was observed between the TP and LP groups. There was no main effect of learners’ prior knowledge for intrinsic cognitive load ratings, *F*(2, 274) = 0.71, *p* = 0.40. A significant interaction was found between the pretraining approaches and learners’ prior knowledge, *F*(2, 274) = 11.60, *p* < 0.01, *^η^р*^2^ = 0.08. There was a significant simple effect of pretraining approaches for higher prior knowledge learners, *F*(2, 274) = 25.22, *p* < 0.01, *^η^р*^2^ = 0.16. Following this significant effect, a Tukey HSD *post hoc* test showed the higher prior knowledge learners in the no pretraining group had significantly higher ratings of intrinsic cognitive load than the higher prior knowledge learners in the linguistic pretraining group (*p* < 0.01) and higher prior knowledge learners in the thematic pretraining group (*p* < 0.01). There was no significant difference between ratings of intrinsic cognitive load for the higher prior knowledge learners in the latter two conditions. No significant simple effect was found for lower prior knowledge learners in terms of intrinsic cognitive load ratings, *F*(2, 274) = 0.09, *p* = 0.92.

A two-way ANOVA found a significant main effect of pretraining approaches on the ratings of extraneous cognitive load, *F*(2, 274) = 6.24, *p* < 0.01, *^η^р*^2^ = 0.04. Both the learners in the TP conditions (*M* = 1.56, SD = 0.68) and the learners in the LP conditions (*M* = 1.71, SD = 0.73) had significantly lower ratings of extraneous cognitive load than the learners in the NP conditions (*M* = 1.98, SD = 0.98). However, no significant difference was observed between the TP and LP groups. There was no main effect of learners’ prior knowledge on the ratings of extraneous cognitive load, *F*(1, 274) = 1.28, *p* = 0.26. A significant interaction was found between the pretraining approaches and learners’ prior knowledge, *F*(2, 274) = 10.79, *p* < 0.01, *^η^р*^2^ = 0.07. A significant simple effect of extraneous cognitive load was found for higher prior knowledge learners, *F*(2, 274) = 16.71, *p* < 0.01, *^η^р*^2^ = 0.11. Following this effect, a Tukey HSD post hoc test showed the higher prior knowledge learners in the no pretraining group experienced significantly higher levels of extraneous cognitive load than the higher prior knowledge learners in the linguistic pretraining group (*p* < 0.01) and the higher prior knowledge learners in the thematic pretraining group (*p* < 0.01), with no significant difference between the latter two groups. No significant simple effect was found for lower prior knowledge learners, *F*(2, 274) = 1.00, *p* = 0.37.

#### Instructional efficiency

Cognitive load ratings are frequently combined with learning performance scores to indicate the relative instructional efficiency for different instructional situations. Instructional efficiency in this research was calculated adopting Pass and van Merrienboer’s (1993) formula *E* = (*P*-*R*)/√2, in which *E* stands for efficiency, *P* for learning performance z-score, and *R* for cognitive load rating z-score. In this study, the cognitive load z-score was calculated using the mean of intrinsic cognitive load and extraneous cognitive load ratings. According to the formula, higher values of instructional efficiency can be achieved in learning environments where learning performance is high while cognitive load is low; lower values of instructional efficiency occur in instructional situations where learning performance is low while cognitive load is high.

A two-way ANOVA showed a significant main effect of learners’ level of prior knowledge, *F*(1, 274) = 34.69, *p* < 0.01, *^η^р*^2^ = 0.11, indicating a small effect size. The higher prior knowledge learners (*M* = +0.39, SD = 1.08) had significantly higher instructional efficiency than the lower prior knowledge learners (*M* = −0.34, SD = 1.15). There was also a significant main effect of pretraining approaches, *F*(1, 274) = 23.31, *p* < 0.01, *^η^р*^2^ = 0.15, showing a small effect size. The learners in the TP conditions (*p* < 0.01) and in the LP conditions (*p* < 0.01) had significantly higher instructional efficiency than the learners in the NP conditions. However, the TP groups did not differ significantly from the LP groups. A significant interaction was found between the pretraining approaches and the levels of learners’ prior knowledge, *F*(2, 274) = 6.08, *p* < 0.01, *^η^р*^2^ = 0.04. There was a significant simple effect for higher prior knowledge learners, *F*(2, 274) = 27.39, *p* < 0.01, *^η^р*^2^ = 0.18, indicating a small effect size. Following this significant effect, a Tukey HSD *post hoc* test showed the higher prior knowledge learners in the no pretraining group had significantly lower instructional efficiency than higher prior knowledge learners in the LP group (*p* < 0.01) and higher prior knowledge learners in the TP group (*p* < 0.01). There was no significant difference in terms of instructional efficiency between the LP group and the TP group. A significant simple effect was also found for the lower prior knowledge learners, *F*(2, 274) = 3.10, *p* < 0.05, *^η^р*^2^ = 0.02. A Tukey HSD post hoc test showed that the learners in the TP group had significantly higher instructional efficiency than the learners in the NP group, *p* < 0.05. However, there were no significant differences between the TP group and LP group or between the LP group and the NP group.

## Discussion

This research investigated the pretraining effect and the expertise reversal effect in learning classical Chinese texts among adolescent Chinese speakers. First, the results supported the pretraining effect as learners in the pretraining conditions (thematic and linguistic pretraining groups) had higher reading comprehension performances and experienced lower cognitive load ratings than the learners in the no pretraining condition. Second, the study highlighted the positive effect of thematic pretraining in facilitating the acquisition of classical Chinese reading skills among the learners with higher prior knowledge. Third, the findings confirmed that there was an interaction between the levels of learners’ prior knowledge and the effectiveness of instructional formats (on inferential test scores, measures of cognitive load and instructional efficiency). Fourth, simple effect tests indicated that higher prior knowledge learners in the TP group had better inferential test scores in classical Chinese reading, experienced lower level of cognitive load, and enjoyed higher instructional efficiency than higher prior knowledge learners in the LP and NP groups. However, learners with lower levels of prior knowledge in the three instructional conditions did not have statistically significant differences in terms of total test scores and sub-test scores. This actually refined and contextualized what had been usually observed for pre-training in cognitive load theory-based studies – pre-training works for novices but not for experts–within a complex, language-specific learning domain, Classical Chinese reading (Clarke et al., 2005; Homer and Plass, 2010). This issue will be addressed later in this discussion.

In this study, both higher and lower prior knowledge learners in the LP and TP instructional conditions had higher post-test scores and lower cognitive load ratings than learners in the NP groups, demonstrating the overall pretraining effect (in line with Mayer et al., 2002; Mayer et al., 2002; Gegner et al., 2009; Fiorella and Mayer, 2012; Pilegard and Mayer, 2016, 2018). This effect means that segmenting complicated cognitive tasks into smaller, isolated individual parts and providing preliminary information on these parts in advance can significantly reduce element interactivity of complete instructional materials at the following stage and help leaners allocate their cognitive resources more effectively, thus improving learning outcomes. Pretraining activities in this study could have facilitated the comprehension process as the participants learned the essential elements (linguistic knowledge and contextual knowledge) in advance, consequently reducing the element interactivity of the instructional materials. Accordingly, the learners in the two pretraining conditions demonstrated better comprehension performance, lower cognitive load ratings, and higher instructional efficiency than the learners who did not receive pretraining.

In addition, the findings that pretraining activities provided more benefits to both higher and lower prior knowledge learners compared to no pretraining conditions were consistent with previous language acquisition studies (Pang et al., 2003; Park, 2004; Past, 2019). Linguistic pretraining activities benefited Chinese classics reading comprehension as vocabulary plays an essential role in acquiring reading skills. Pang et al. (2003) claimed that vocabulary could be taught directly while “giving word definitions and pre-teaching of vocabulary before reading a text” (p. 12). This view was also shared by Nation (2008) and Past (2019). Past’s (2019) study confirmed that vocabulary pretraining could improve learners’ reading comprehension. Similarly, Grabe and Stroller (2011) also argued that reading comprehension became easier once learners had more prior vocabulary knowledge related to the instructional material.

Pretraining could also be provided in the form of graphics, pictures, or comic strips. The thematic pretraining materials in this research, comic strips, could equip learners with background information about the instructional text and develop learners’ mental models. The thematic pretraining materials could prepare learners with contextual information to build the connections between their prior knowledge and the instructional contents, which is consistent with previous research studies (Echevarría et al., 2008; Park, 2004; Rance-Roney, 2010). For example, Rance-Roney (2010) also suggested instructors to use “prereading videos and photographs related to the unit theme to prepare the learners with visual and auditory input that would ready them for challenging text” (p. 387). Similarly, Huang (2019) found that sequential visual aids could indicate the storyline of the learning text, thus facilitating Chinese learners’ comprehension. Short and Fitzsimmons (2007) claimed that reading teachers should attach great importance to background information and cultural knowledge in facilitating learners’ reading comprehension.

It is also worth noticing that the thematic pretraining benefited lower prior knowledge learners more than the linguistic pretraining did. The results indicated that the thematic pretraining, which targeted learners’ higher-level reading skills, also facilitated lower prior knowledge students’ overall performance. It could not be denied that lower-level skills like vocabulary recognition and retrieval play a critical role in the process of turning controlled processing into automatic processing, but it might be difficult for students to fully master low-level skills such as vocabulary for Classical Chinese reading. When students were still struggling with classical Chinese reading, it would be more beneficial to give them thematic pretraining, such as the basic storyline of the text, to facilitate deeper learning. This aligns with Uebergang et al. (2024) who believed that learners who had learning difficulties may still “rely on visual modes of representation to make meaning with texts” and gain “benefit from visual representations to scaffold their reading of printed text” (p. 34). In a similar vein, McVicker (2007, 2018) argued that supplementary visual aids such as comic strips could scaffold text structures so that learners could rely on the visual clues to deduce or infer the information delivered in the instructional materials.

Moreover, an expertise reversal effect was found as higher prior knowledge learners gained more benefits from thematic and linguistic pretraining activities, with thematic pretraining enjoying an advantageous edge, than lower prior knowledge learners (Jiang et al., 2018; Jiang, 2024; Kalyuga et al., 2003). This result actually manifests a refinement to that found in previous cognitive load theory-based research according to which pretraining activities were more effective for less knowledgeable learners than for more knowledgeable learners (Clarke et al., 2005; Homer and Plass, 2010). One plausible explanation could be that the more proficient learners in this research could not be regarded as experts but just relatively more knowledgeable novices. Even though they had learned Chinese as the first language in formal school settings for 10 years, they still could be regarded as novices in classical Chinese reading, because classical Chinese texts constitute a small proportion of the school curriculum.

Another explanation comes from the measures of cognitive load. While pretraining reduced cognitive load for “experts” (learners with higher prior knowledge in this study), it did not for “novices” (learners with lower prior knowledge), which extends traditional cognitive load theory’s patterns. After receiving the thematic pretraining, more knowledgeable learners in the TP group would construct the textual structure as a mental model that can be used in the reading stage to bridge gaps when essential information cannot be easily inferred or not totally given in the text. Gegenfurtner et al. (2011) argued that higher expertise learners were more able to focus on the relevant elements of visual input and ignore the irrelevant parts. After reading the thematic pretraining comic strips, the learners in the TP conditions should construct the schematic visualizations which “reduce the number of cues and depict only relevant information in abstract, simplified form” (Gegenfurtner et al., 2011, p. 526). To this end, more knowledgeable learners were more able to discern the relevant information and carry on relevant cues to schematic construction, thus experiencing lower levels of cognitive load (Woolley, 2014). This aligns with Huang’s (2019) research which confirmed that visual aids benefited intermediate Chinese as a foreign language learners’ reading comprehension.

Research has shown that integrating visual and textual information facilitates mental model construction (Butcher, 2006). Gegenfurtner et al. (2011) found that high expertise individuals had slightly longer eye fixation durations on task-relevant sections and shorter eye fixations on task-redundant sections than low expertise individuals. Brumberger (2022) reported that high expertise learners processed visual materials more efficiently and effectively than low expertise learners, though the differences were far less extensive.

Higher prior knowledge learners also gained more benefits from the LP instructional condition than lower prior knowledge learners. It was hypothesized that providing linguistic pretraining in the form of learning essential words and phrases in advance could not only help readers engage in decoding processes at the phonological-semantic level by applying their linguistic knowledge, but also assist them in engaging in orthographic-syntactic processing and discursive-contextual processing (Anderson and Pearson, 1984; Graesser, 2007; Lesgold and Perfetti, 1978; McVee et al., 2005; Ruddell and Unrau, 2013). However, presenting the essential linguistic components in separable phases may also cause split attention situations. This could possibly explain why cognitive load ratings of the lower prior knowledge learners were not reduced, as they had less cognitive resources to integrate pretrained linguistic information and textual information in the latter phase to generate a coherent representation of meaning.

The major pedagogical implication of this research is that providing pretraining activities could facilitate classical Chinese reading comprehension, alleviate cognitive load, and improve instructional efficiency for Chinese adolescent learners. Furthermore, thematic pretraining activities could be more advantageous than linguistic pretraining activities for relatively higher prior knowledge learners as they may already have sufficient prior knowledge to integrate the contents of visual and textual information and build the connections between their prior knowledge and novel information. It should be noted that lower prior knowledge learners also favored thematic pretraining, though no statistical differences between the two pretraining formats were observed for these learners.

As the first attempt to apply pretraining principle in classical Chinese reading instruction, the reported research has several limitations that should be acknowledged. First, this study simply assumed that higher year level students had more prior knowledge related to classical Chinese reading than lower year level students. Future studies need to use pretests to accurately assess participants’ levels of expertise. Second, the quasi-experimental design used in this study may have affected the validity of the research findings. Future research should adopt random sampling when allocating research participants into experimental conditions. Third, although the instructional materials were designed to reduce split-attention effects, the use of annotations presented in side text boxes may still have imposed additional extraneous cognitive load, particularly for learners with lower prior knowledge. Future studies could explore more integrated presentation formats or adaptive annotation strategies to further minimize potential split-attention effects. Fourth, even though the effect of thematic pretraining in the form of comic strips has promoted classical Chinese learning, future studies could explore the effectiveness of other forms of thematic and linguistic pretraining materials, like colored or monochrome visualizations, animated or live actions, to find out the best combinations of presentations to facilitate classical Chinese learning.

## Conclusion

This research study offers empirical evidence supporting the role of pretraining in managing cognitive load and facilitating the acquisition of Classical Chinese reading skills. Grounded in cognitive load theory, our investigation found that both linguistic and thematic pretraining can reduce cognitive load and improve comprehension when learners engage with authentic, high-interactivity texts. The distinction between these pretraining types—with linguistic pretraining supporting foundational decoding and thematic pretraining aiding higher-level meaning integration—proves valuable for instructional design. By applying the pretraining effect and expertise reversal effect of cognitive load theory to the challenging domain of Classical Chinese, our work provides a relevant perspective for educational psychology and language education research.

## Data Availability

The datasets presented in this article are not readily available because the data is currently being used for ongoing research and is not available for external sharing at this time. Requests to access the datasets should be directed to Dayu Jiang, d.jiang@whu.edu.cn.

## Appendix 1

### Subjective rating of cognitive load

All of the following questions refer to the teaching activity that just finished. Please respond to each of the questions on the following scale (1 meaning NOT AT ALL THE CASE and 5 meaning COMPLETELY THE CASE).

**Table:**

Item	1	2	3	4	5
The whole teaching was very complex.	1	2	3	4	5
The pretraining was very complex.	1	2	3	4	5
Vocabulary learning in the teaching material was very complex.	1	2	3	4	5
Sentence learning in the teaching material was very complex.	1	2	3	4	5
Content learning in the teaching material was very complex.	1	2	3	4	5
This session is not effective for classical Chinese learning.	1	2	3	4	5
The pretraining materials was not effective for learning.	1	2	3	4	5
The teaching about vocabulary learning was unclear.	1	2	3	4	5
The teaching about sentence learning was unclear.	1	2	3	4	5
The teaching about content learning was unclear.	1	2	3	4	5
